# Personal Activity Intelligence and Ischemic Heart Disease in a Healthy Population: China Kadoorie Biobank Study

**DOI:** 10.3390/jcm11216552

**Published:** 2022-11-04

**Authors:** Pål Hammer, Atefe R. Tari, Barry A. Franklin, Chi-Pang Wen, Ulrik Wisløff, Javaid Nauman

**Affiliations:** 1 Department of Circulation and Medical Imaging, Faculty of Medicine and Health Sciences, Norwegian University of Science and Technology, 7491 Trondheim, Norway; 2 Department of Neurology and Clinical Neurophysiology, St. Olavs Hospital, Trondheim University Hospital, 7006 Trondheim, Norway; 3 Healthy Living for Pandemic Event Protection (HL-PIVOT) Network, Chicago, IL 60612, USA; 4 Preventive Cardiology and Cardiac Rehabilitation, William Beaumont Hospital, Royal Oak, MI 48073, USA; 5 William Beaumont School of Medicine, Oakland University, Rochester, MI 48309, USA; 6 National Health Research Institute, Zhunan Town 35053, Taiwan; 7 Graduate Institute of Biomedical Sciences, College of Medicine, China Medical University, Taichung City 40447, Taiwan; 8 School of Human Movement and Nutrition Science, University of Queensland, St. Lucia, QLD 4072, Australia; 9 Institute of Public Health, College of Medicine and Health Sciences, United Arab Emirates University, Al Ain 15551, United Arab Emirates

**Keywords:** physical activity, exercise, ischemic heart disease, myocardial infarction, activity metric, Personal Activity Intelligence

## Abstract

Background: Personal Activity Intelligence (PAI) is a physical activity metric that translates heart rate during physical activity into a simple score, where a weekly score of 100 or greater is associated with a lower risk of cardiovascular disease and mortality. Here, we prospectively investigated the association between PAI and ischemic heart disease (IHD) mortality in a large healthy population from China. Methods: Using data from the China Kadoorie Biobank, we studied 443,792 healthy adults (60% women). The weekly PAI score of each participant was estimated based on the questionnaire data and divided into four groups (PAI scores of 0, ≤50, 51–99, or ≥100). Adjusted hazard ratios (aHRs) and 95% confidence intervals (CIs) for fatal IHD and nonfatal myocardial infraction (MI) related to PAI were estimated using Cox proportional hazard regression analyses. Results: There were 3050 IHD deaths and 1808 MI events during a median follow-up of 8.2 years (interquartile range, 7.3–9.1; 3.6 million person-years). After adjustments for multiple confounders, a weekly PAI score ≥ 100 was associated with a lower risk of IHD (aHR: 0.91 (95% CI: 0.83–1.00)), compared with the inactive group (0 PAI). The corresponding aHR for MI was 0.94 (95% CI: 0.83–1.05). In participants aged 60 years or older at baseline, the aHR associated with a weekly PAI score ≥ 100 was 0.84 (95% CI, 0.75–0.93) for IHD and 0.84 (95% CI, 0.73–0.98) for MI. Conclusion: Among healthy Chinese adults, a weekly PAI score of 100 or greater was associated with a lower risk of IHD mortality across all age groups; moreover, a high PAI score significantly lowered the risk of MI but only in those 60 years and older at baseline. The present findings extend the scientific evidence that PAI may have prognostic significance in diverse settings for IHD outcomes and suggest that the PAI metric may be useful in delineating the magnitude of weekly physical activity needed to reduce the risk of IHD mortality.

## 1. Introduction

Ischemic heart disease (IHD) is the leading cause of cardiovascular disease (CVD) mortality and the preponderant contributor to disease burden and mortality worldwide. The prevalence of IHD increased from <100 million in 1990 to 197 million in 2019, and the estimated global IHD deaths increased from 5.69 to 9.14 million during this period [1]. The age-standardized death rates for IHD are increasing in many parts of the United States, the United Kingdom, and China. For example, age-standardized IHD death rates in China increased from 110 per 100,000 in 1990 to 138 per 100,000 in 2016 [2]. Accordingly, countries and health systems need to increasingly focus on delivering effective preventive interventions to potentially reverse the alarming trends in morbidity and mortality secondary to IHD [1].

The health benefits of regular physical activity (PA) and the dose–response relationship between the amount of PA and CVD morbidity and mortality are well established [3,4]. However, recent estimates suggest that ~32% of women and ~23% of men worldwide were not meeting the recommended levels of weekly PA (i.e., 150 to 300 min of moderate-intensity PA, 75 to 150 min of vigorous-intensity PA, or combinations thereof) for health benefits [5]. Although the global prevalence of insufficient PA was relatively stable between 2001 and 2016, a considerable variation in trends across regions, income groups, and countries was observed with the largest increases in physical inactivity in high-income countries and the greatest decreases in East and Southeast Asia [5]. Nevertheless, only 23% of Chinese adults between the ages of 20 and 59 were meeting the recommended levels of PA in 2014 [6]. The commonly reported barriers of PA participation include lack of time, inability to self-manage such as setting personal goals and monitoring PA progress through personalized feedback, and competing domestic and occupational responsibilities [7,8]. In addition, a positive association between leisure-time PA and socioeconomic status is well recognized, and individuals of a high socioeconomic status are reported to be more physically active than those of a lower socioeconomic status [9]. Furthermore, movement restrictions and lockdowns during the COVID-19 pandemic have resulted in decreases in PA and increases in sedentary time [10].

Given the pandemic scale of physical inactivity [11], innovative methodologies and interventions to increase PA participation are sorely needed, and modern healthcare technologies can be used to improve PA uptake and overcome common barriers to PA participation [12,13,14]. The COVID-19 disease may be associated with various CVD pathologies, and CVD patients infected with COVID-19 are reported to have the worst outcomes and an increased risk of mortality [15]. Therefore, appropriate health care technologies including wearable devices, web applications and platforms, and tele-rehabilitation could help to achieve sustainable patient engagement and may lead to more favorable health outcomes [16]. Personal Activity Intelligence (PAI) is a metabolic PA metric that considers the age, sex, resting and maximal heart rate, and heart rate response to PA of individuals. PAI was developed to objectively quantitate a physically active lifestyle and has now been integrated into self-assessment heart rate devices and/or health apps. It translates heart rate variations over the course of a week into a simple and easily understandable score (0 PAI = inactive and 100 PAI = sufficiently active) [17,18,19].

The predictive ability of PAI for varied health outcomes across diverse settings has been previously reported [17,18,20,21,22,23,24,25,26]. Briefly, a weekly PAI score of ≥100 was associated with a lower risk of all-cause and CVD mortality in cohorts of relatively healthy individuals [17,18,21] and in subgroups of patients with CVD [24]. Moreover, a sustained high PAI score and an increase in PAI over time was associated with a reduced risk of all-cause and CVD mortality [22,23]. We also found that maintaining a weekly PAI score ≥ 100 was associated with significant reductions in the incidence of dementia [26]. Furthermore, in earlier studies conducted before the WHO 2020 PA guidelines, the PAI metric predictive ability was superior in meeting the PA recommendations (minimum of 150 weekly minutes of moderate-intensity PA or a minimum of 75 weekly minutes of vigorous-intensity PA) where individuals categorized into a group with a PAI score ≥ 100 and not meeting the PA recommendations had a similar mortality risk compared with the individuals with a PAI score ≥ 100 and meeting the PA recommendations. However, individuals meeting the PA recommendations but not obtaining a PAI score ≥ 100 had higher mortality risks [18,24]. The PAI metric has been shown to fit well with the new PA guidelines (150 to 300 weekly minutes of moderate-intensity PA or 75 to 150 weekly minutes of vigorous-intensity PA). This is particularly the case with the upper limits of the recommendations where individuals obtaining ≥100 PAI without meeting the upper limit of the PA recommendations or those meeting the PA recommendations but not obtaining ≥100 PAI had similar mortality risks as the reference cohort of individuals with ≥100 PAI and meeting the PA recommendations [21]. To our knowledge, only one previous study, conducted in the U.S., reported on the association between PAI and IHD using the Aerobics Center Longitudinal Study database [21], and the findings show that a weekly PAI score ≥ 100 was associated with a lower risk of IHD mortality. The present study was undertaken to evaluate the association between PAI and IHD using the China Kadoorie Biobank, a large prospective cohort of Chinese adults.

## 2. Materials and Methods

### 2.1. Study Participants

The China Kadoorie Biobank is a nationwide, prospective cohort study from 10 geographically diverse regions across China. Participants were chosen from Disease Surveillance Points (DSP) which is a nationally representative system [27,28], and study areas were selected based on levels of economic development, local disease patterns, various levels of risk exposures, quality of outcome reporting systems, and local commitment to the project. Details of study design and methods have been previously described [29,30]. Briefly, participants were invited to study clinics between June 2004 and July 2008, and a total of 512,714 individuals, 30–79 years of age, were eligible for inclusion. We wanted to investigate the association between PAI and IHD in relatively healthy participants at baseline; therefore, we excluded participants with a previous diagnosis of IHD and/or stroke (*n* = 23,129), cancer (*n* = 2385), chronic obstructive pulmonary disease (*n* = 34,541), tuberculosis (*n* = 5738), those who reported any fracture at baseline examination (*n* = 1114), and those with missing data on PA habits (*n* = 2015). The final analyses included 443,792 participants (177,529 men and 266,263 women) (Supplementary Figure S1). All participants provided written informed consent. The study was conducted in accordance with the Declaration of Helsinki and approved by the ethics committees or institutional review boards at the University of Oxford, the Chinese Center for Disease Control and Prevention (China CDC), the Chinese Academy of Medical Sciences, and relevant participating regions (DAR-2019-00063; 17 December 2019) for studies involving humans.

### 2.2. Clinical Measurements and Questionnaire-Based Information

Trained staff measured body weight, height, and blood pressure using standardized methods with calibrated instruments. After ≥5 min of rest in a seated position, blood pressure was measured at least twice using an automated digital monitor (model UA-779, A&D Medical), and the mean of 2 satisfactory measurements was used for analyses [31]. Stepwise on-site testing of plasma glucose level was determined using the SureStep Plus System (Johnson & Johnson, New Brunswick, NJ, USA). Self-reported data on sociodemographic characteristics (age, sex, occupation, household income, education, and marital status), lifestyle behaviors (PA, smoking, and alcohol consumption), personal and medical history (diabetes, hypertension, and general health), and family history of CVD were obtained at baseline through standardized questionnaires [30].

### 2.3. Personal Activity Intelligence

Information on the frequency and duration of leisure-time PA was obtained via a self-reported questionnaire based on 6 specific activities: Tai chi/Qigong, walking, jogging/aerobic exercise, swimming, ball games (basketball, table tennis, etc.), and other activities (e.g., mountain climbing). The response options to the frequency of PA by participants such as “1–2 times/week”, “3–5 times/week”, and “daily or almost every day” were translated to 1.5, 4, and 5 week days, respectively. The question “Approximately how many hours per week did you perform exercise during your leisure time” was used to determine the duration of PA. The intensity of PA was classified by activity-specific metabolic equivalents (METs; 1 MET = 3.5 mLO_2_/kg/min) available from the Compendium of Physical Activities [32,33]. We calculated the PAI score of each participant based on the frequency, duration, and intensity of leisure-time PA. According to the PAI algorithm previously described [18,19], weekly minutes of PA were calculated by multiplying the average frequency and duration, and the strenuousness of physical exertion was translated into a relative PA intensity using ~44%, 73%, and 83% of heart rate reserve for low-, moderate-, and vigorous-intensity activities, respectively. Exercise volumes were then combined with intensity calculations, using the heart rate reserve, to estimate the weekly PAI score which was based on the questionnaire data.

### 2.4. End-Points and Follow-Up

The health outcomes of all participants were obtained using linkage with DSP system, the national health insurance claim system and through established local chronic disease registries [27,28]. The underlying causes of death were coded using the 10th International Classification of Diseases (ICD) by trained staff, who were blinded to baseline information. The primary outcome in the analyses was fatal IHD (ICD10: I20-I25), and the secondary outcome was nonfatal myocardial infarction (MI) (ICD10: I21-I23). Study participants were followed from baseline to date of event, loss to follow-up, or to 1 January 2015, whichever came first.

### 2.5. Statistical Analyses

Baseline characteristics of the participants were compared using Cochran–Armitage test for categorical variables and regression analyses for continuous variables, and data are presented as number (%) for categorical variables and mean (SD) for continuous variables. Participants were categorized into 4 groups according to their weekly PAI score: 0 (inactive), ≤50, 51–99, or ≥100 [17,18]. The inactive group (0 PAI) was used as a reference cohort. To investigate the association between PAI and IHD/MI, we used stratified Cox proportional hazard models. The first model included age (as the underlying time scale), year of study stratified by sex, study area, and baseline age in 5-year intervals. The final model additionally included body mass index (<18.5, 18.5–24.9, 25–29.9, or ≥30 kg/m^2^), hypertension (yes, no), diabetes status (yes, no), smoking history (never, previous, or current), education (no formal education, primary school, middle or high school, technical school/college, or university), alcohol consumption (average grams of alcohol per week), self-rated general health (poor, fair, good, or excellent), marital status (single, married, widow/er, or separated/divorce), occupation (agriculture and related workers, factory worker, administrator/manager, professional/technical, sales and service workers, house wife/husband, self-employed, unemployed, retired, or other/not stated), household income, and family history of CVD (yes, no) [17]. The assumption of proportional hazards was examined and satisfied using Schoenfeld residuals, and results are reported as adjusted hazard ratios (aHRs), and 95% confidence intervals (CIs) indicate the precision of estimates.

We also investigated the associations of PAI with IHD in prespecified subgroups of participants based on baseline age, and those with known IHD risk factors, such as smoking, hypertension, diabetes, or overweight/obesity. In a separate analysis, study participants were categorized into <100 PAI and ≥100 PAI, and we assessed the association of PAI with IHD and MI. All statistical tests were 2 sided, and *p* < 0.05 was considered significant. The statistical analyses were performed using Stata for Windows (version 16, StataCorp LLC, College Station, TX, USA).

## 3. Results

The characteristics of study participants according to their weekly PAI scores are presented in Table 1. Among the 443,792 participants, 22.7% attained a weekly PAI score of ≥100. These individuals tended to be older, with higher levels of education and were more likely to have “excellent” or “good” self-reported health status compared with their inactive counterparts.

The baseline characteristics of individuals are also presented both for IHD and MI status and according to the weekly PAI scores in the Supplementary Tables S1 and S2. Regardless of the event status at follow-up, those with a weekly PAI score of ≥100 had higher levels of education and reported “excellent/good” health status at baseline compared with the inactive cohort.

There were 3050 IHD deaths and 1808 MI events during a median follow-up of 8.2 years (interquartile range, 7.3–9.1; 3.6 million person-years). After adjustments for multiple confounders, a weekly PAI score ≥ 100 was associated with a lower risk of IHD (aHR: 0.91 (95% CI: 0.83–1.00)) compared with the inactive group (0 PAI). The corresponding aHR for MI was 0.94 (95% CI: 0.83–1.05). Among participants with a weekly PAI score < 100 as reference, the aHRs associated with ≥100 PAI were 0.91 (95% CI: 0.83–1.00) for IHD and 0.93 (95% CI: 0.83–1.05) for MI (Table 2 and Table 3).

**Figure 1:**
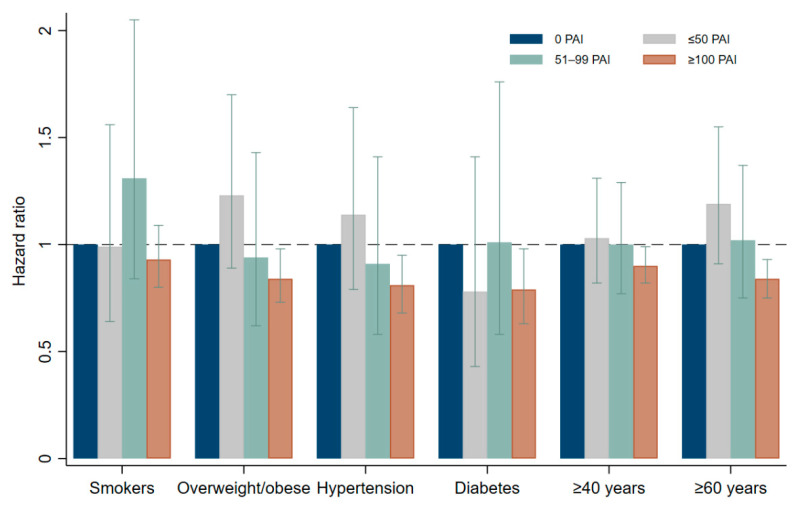
Hazard ratio of ischemic heart disease mortality associated with Personal Activity Intelligence (PAI) in subgroups of participants. Rectangular bars show the hazard ratios, and error bars represent the 95% confidence interval. Smokers: *n* = 110,622 with 663 and 290 events in inactive and ≥100 PAI groups, respectively. Overweight/obese: *n* = 146,128 with 619 and 370 events in inactive and ≥100 PAI groups, respectively. Hypertension: *n* = 57,364 with 590 and 329 events in inactive and ≥100 PAI groups, respectively. Diabetes: *n* = 23,688 with 283 and 212 events in inactive and ≥100 PAI groups, respectively. ≥40 years: *n* = 362,616 with 1899 and 944 events in inactive and ≥100 PAI groups, respectively. ≥60 years: *n* = 86,484 with 1267 and 702 events in inactive and ≥100 PAI groups, respectively.

The stratified analyses show an effect modification by age at the cutoffs of 40 and 60 years for IHD and MI outcomes (*p* < 0.01). Compared with the reference cohort of 0 PAI, the aHRs of IHD associated with a weekly PAI score ≥ 100 was 0.90 (95% CI, 0.82–0.98) in participants older than 40 years and 0.84 (95% CI, 0.75–0.93) in participants who were older than 60 years at baseline (Figure 1). The aHRs of MI associated with a weekly PAI score of ≥100 was 0.92 (95% CI, 0.81–1.04) for those older than 40 years and 0.84 (95% CI, 0.73–0.98) in participants older than 60 years at baseline (data not shown).

In other subgroups of participants, a weekly PAI score ≥ 100 was associated with a lower risk of IHD mortality. For example, compared with inactive hypertensive participants, the aHR for hypertensive participants with a PAI ≥ 100 was 0.81 (95% CI, 0.68–0.95). Similarly, the aHRs associated with a PAI ≥ 100 was 0.79 (95% CI, 0.63–0.98) in patients with diabetes and 0.84 (95% CI, 0.73–0.98) in overweight/obese individuals (Figure 1).

## 4. Discussion

In this large prospective cohort of healthy men and women in China, we found that a weekly PAI score of greater than or equal to 100 was associated with a lower risk of IHD across all age groups and associated risk factor profiles. A high PAI score also significantly lowered the risk of MI but only in those 60 years and older at baseline.

The present findings are consistent with the results of the Aerobics Center Longitudinal study showing that a weekly PAI score of ≥100 is associated with lower risk of IHD mortality [21]. In the study of 56,175 relatively healthy participants at baseline, a weekly PAI score ≥ 100 was associated with a 30% lower risk of IHD mortality compared with an inactive reference cohort [21]. These results suggest that PAI has prognostic significance for IHD outcomes in diverse settings, including high- and middle-income countries with varied ethnic populations.

To our knowledge, this is the first study to assess the association between PAI and MI outcomes. Although high weekly PAI scores were associated with a lower risk of MI compared with the inactive or reference cohort, statistical significance was only achieved in those who were 60 years or older. The nonsignificant results in the total cohort may partly be explained by the very low incidence of MI events during the follow-up period, thus restricting the statistical power of the analysis and affecting the precision of the estimates. Furthermore, the significant association between PAI and MI in those 60 years or older may partly be due to an increase in the PA levels in the Chinese elderly population [34]. Nevertheless, previous studies related to PA and the risk of MI have also reported an inverse relation between high levels of PA and the risk of subsequent MI [35,36]. A meta-analysis of 33 studies showed that moving from an inactive state to moderate PA was associated with 20% lower risk of IHD incidence and mortality, and even low levels of PA (less than 11.5 METs h/week) were associated with favorable outcomes [35]. Similarly, the results from a large cohort of general population followed up for an average of 9 years demonstrated that high levels of PA were associated with a lower risk of acute MI (HR: 0.66, 95% CI: 0.50–0.89) and IHD (HR: 0.49, 95% CI: 0.38-0.64) [36].

We observed a 10% to 21% lower risk of IHD mortality associated with a weekly PAI score ≥ 100 across individuals stratified according to hypertension, body mass index, or diabetes. These results suggest that “at risk” subgroups of patients may reduce their risk of IHD mortality by achieving high weekly PAI scores. Previous studies in hypertensive patients have also shown a beneficial effect of high levels of PA for CVD mortality. For instance, moderate or high levels of PA were associated with a lower risk of CVD events across all levels of blood pressure (HRs of 0.81, 0.69, and 0.77 for pre-, stage 1, and stage 2 hypertension, respectively) [37]. The results of the prospective cohort of 5859 individuals with diabetes at baseline showed that moderate levels of PA were associated with a lower risk of CVD mortality (HR: 0.51, 95% CI: 0.32–0.81), and the findings of the meta-analysis were consistent, showing a lower risk of CVD mortality associated with high levels of PA (HR: 0.63, 95% CI, 0.48–0.83) [38]. For MI outcomes, we observed similar trends as for IHD; however, the statistical power of these analyses was limited due to fewer events in the subgroups of participants (data not shown).

The burden of IHD has continued to increase in China since 2000 and accounted for ~40% of all CVD mortality in 2019 [39]. Although participation in PA has increased over the years, only 23% of Chinese adults were meeting contemporary PA recommendations in 2014 [6]. Emerging healthcare technologies could be used as facilitators for health behavior change [14,40]. For example, wearable devices promoting a physically active lifestyle could help individuals to track and self-monitor their PA. The PAI metric is integrated into wearable devices with a downloadable application that is freely available worldwide. Recent studies using objective measurements of PAI through a wearable heart rate monitor and mobile app have shown that individuals with type 2 diabetes using PAI significantly improved their exercise capacity and sleep time when compared with the control group following current PA recommendations [41]. Similarly, the monitoring of PAI scores was associated with an increase in PA among cardiac patients compared with those not using PAI [42]. The advantages of PAI may be partially attributed to the personalized metric it provides based on the individual heart rate response to exercise, which provides a readily available index of exercise intensity and energy expenditure. These data may be shared between patients and their healthcare providers and offer an opportunity for physicians to motivate and empower their patients to achieve a cardioprotective weekly PAI score.

The strengths of the present study include a large population-based cohort of apparently healthy men and women, comprehensive information on participant risk factors for cardiovascular mortality, and an 8.2-year average follow-up. Moreover, using the same cut-off values of PAI as used in the present study, the findings of prospective studies from Norway [19], China [17], and the U.S. [21] showed an inverse association between PAI and all-cause mortality and CVD outcomes. These results strengthen the reliability and validity of the PAI metric across various ethnicities and socioeconomic strata and suggest that PAI has prognostic implications in diverse settings. Nevertheless, we acknowledge some limitations of our study methodology. First, due to the observational nature of our study, the findings are not necessarily causal. Second, the estimation of PAI was based on self-reported data which are prone to information bias. However, due to our prospective study design, measurement errors and the nature of misclassification are most likely to be nondifferential, and the measures of association are more likely to be biased towards the null. Third, residual unmeasured and unknown factors such as prescribed medications and dietary practices, which were unaccounted for in the analyses, may have influenced our estimates. Indeed, healthy dietary patterns consisting of diets high in vegetables, whole grains, fibers, and legumes and low in red and processed meat, as well as an active lifestyle, are shown to be associated with a lower risk of IHD [43,44]. Finally, although the China Kadoorie Biobank is a nationwide representative sample of Chinese adults, and the PAI metric has previously been shown to predict IHD mortality in a large US cohort [21], additional confirmatory studies of PAI across disparate races and ethnicities are needed before the present findings can be extrapolated to other populations in low-, middle-, and high-income countries.

Modern wearable health technologies have the potential to revolutionize health management based on the notion that the self-tracking of health behavior leads to self-knowledge and could potentially provide consumers with direct access to personal analytics that can facilitate preventive care and contribute to their health by aiding in the management of ongoing illness [40,45]. The findings of a recent systematic review and meta-analysis demonstrated that a wearable sensor-assisted home-based cardiac rehabilitation program significantly improved the cardiorespiratory fitness of patients with CVD when compared to center-based cardiac rehabilitation programs [46]. Of note, data related to PA wearables and health outcomes in the general population remain scarce. However, this kind of research is more feasible now than ever largely because of the increased popularity and accessibility of activity-based wearables including accelerometers and should be a goal for future studies. The PAI studies using objective measurements in individuals with diabetes and cardiac patients have shown that the use of PAI is feasible, acceptable and efficacious, and the majority of participants reported increased motivation to exercise and to continue to use PAI long-term [41,42]. Nevertheless, future prospective cohort studies using the objective measurements of PAI are warranted and would help to enhance our understanding of the utility of the PAI score in disease prevention and management. Another important aspect of the behavior change is focusing on patient perception of the risk factors of illness, and the data show that patients seem to underestimate the role of the actual risk factors [47]. Therefore, an increased awareness about the role of various risk factors for health and disease and the promotion of a healthy lifestyle for the prevention and management of illness should be an essential part of public health and clinical settings.

## 5. Conclusions

In summary, PAI was inversely associated with IHD outcomes in this large, prospective study of relatively healthy individuals. Our findings related to the beneficial effect of PA for MI outcomes in individuals 60 years or older are of particular interest, highlighting the importance of moderate-to-vigorous PA for disease prevention in this subgroup, given that the incidence and severity of CVD are proportional to advancing age. These results may partly be explained by the increased PA in the escalating population of older adults in China [36] and suggest that remaining active during the transition from middle-to-older ages is possible and confers significant cardiovascular and survival benefits. Our findings also suggest that the PAI metric may be useful in delineating the magnitude of weekly PA needed to reduce the risk of IHD mortality.

## Figures and Tables

**Table 1:** Baseline characteristics of study participants (*n* = 443,792).

	Inactive (*n* = 324,166)	≤50 (*n* = 10,393)	51–99 (*n* = 8420)	≥100 (*n* = 100,813)	*p*-Value ^a^
Female sex, no. (%)	199,490 (61.5)	6183 (59.5)	5355 (63.6)	55,235 (54.8)	<0.001
Age, mean (SD), y	49.9 (10.1)	50.7 (10.1)	51.3 (10.4)	54.3 (10.6)	<0.001
Urban residence, no. (%)	116,708 (36.0)	7222 (69.5)	4958 (58.9)	64,227 (63.7)	<0.001
Body mass index, no. (%)					
<18.5	13,541 (4.2)	296 (2.9)	219 (2.6)	3331 (3.3)	
18.5–24.9	207,932 (64.1)	6159 (59.3)	5156 (61.2)	61,030 (60.5)	
25.0–29.9	90,390 (27.9)	3445 (33.2)	2680 (31.8)	31,960 (31.7)	
≥30.0	12,303 (3.8)	493 (4.7)	365 (4.3)	4492 (4.5)	<0.001
Systolic blood pressure, mean (SD), mmHg					
	130.3 (20.9)	128.2 (20.8)	129.7 (21.5)	131.1 (21.0)	<0.001
Diastolic blood pressure, mean (SD), mmHg					
	77.8 (11.1)	77.0 (11.2)	77.1 (11.3)	77.3 (11.0)	<0.001
Education level, no. (%)					
No formal education	66,015 (20.4)	731 (7.0)	1088 (12.9)	12,295 (12.2)	
Primary school	107,386 (33.1)	2051 (19.7)	1976 (23.5)	28,579 (28.4)	
Middle or high school	137,522 (42.2)	6058 (58.3)	4219 (50.1)	50,375 (50.0)	
College or university	13,243 (4.1)	1553 (14.9)	1137 (13.5)	9564 (9.5)	<0.001
Smoking status, no. (%)					
Never	224,025 (69.1)	7366 (70.9)	6170 (73.3)	67,623 (67.1)	
Former	17,907 (5.5)	745 (7.2)	593 (7.0)	8741 (8.7)	
Current	82,234 (25.4)	2282 (22.0)	1657 (19.7)	24,449 (24.2)	<0.001
Regular alcohol intake, no. (%)					
Yes	47,952 (14.8)	1592 (15.3)	1142 (13.6)	15,565 (15.4)	<0.001
Household income, yuan/yr, no. (%) ^b^					
<10,000	93,839 (29.0)	2045 (19.7)	2271 (27.0)	23,524 (23.3)	
10,000–19,999	94,746 (29.2)	3139 (30.2)	2354 (28.0)	28,857 (28.6)	
20,000–34,999	78,084 (24.1)	2821 (27.1)	2007 (23.8)	28,784 (28.6)	
≥35,000	57,497 (17.7)	2388 (23.0)	1788 (21.2)	19,648 (19.5)	<0.001
Self-rated health status, no. (%)					
Excellent	60,024 (18.5)	2012 (19.4)	1575 (18.7)	19,405 (19.3)	
Good/Fair	235,365 (72.6)	7526 (72.4)	6071 (72.1)	74,414 (73.8)	
Poor	28,777 (8.9)	855 (8.2)	774 (9.2)	6994 (6.9)	<0.001
Family history of CVD, no. (%)					
Yes	63,128 (19.5)	2518 (24.2)	2018 (24.0)	21,731 (21.6)	<0.001

^a^ For linear trend, regression analyses were used for continuous variables; Cochran–Armitage test was used for proportions of categorical variables. ^b^ At the exchange rate of February 2022, 1 yuan was approximately equal to USD 0.16 or GBP 0.12.

**Table 2:** Hazard ratios of ischemic heart disease mortality by PAI.

PAI	Person-Years	Deaths	HR	(95% CI) ^a^	HR	(95% CI) ^b^
Inactive	2,653,654	1949	1.00	(Ref.)	1.00	(Ref.)
≤50	81,979	76	1.01	(0.80–1.27)	1.02	(0.81–1.29)
51–99	67,421	63	0.95	(0.74–1.23)	0.99	(0.76–1.27)
≥100	805,145	962	0.89	(0.82–0.98)	0.91	(0.83–1.00)
<100	2,803,054	2088	1.00	(Ref.)	1.00	(Ref.)
≥100	805,145	962	0.90	(0.82–0.98)	0.91	(0.83–1.00)

CI, confidence interval; HR, hazard ratio; ischemic heart disease (ICD10: I20-I25); PAI, Personal Activity Intelligence. ^a^ Adjusted for age as time scale, year of study, and jointly stratified by sex, study area, and baseline age in 5-year intervals. ^b^ Adjusted for age as time scale, year of study, BMI (<18.5, 18.5–24.9, 25–29.9, or ≥30 kg/m^2^), smoking (never, previous, or current), alcohol consumption (average grams of alcohol in a typical week), education (no formal education, primary school, middle or high school, technical school/college, or university), household income, hypertension (yes, no), diabetes status (yes, no), marital status (single, married, widow/er, or separated/divorce), self-rated health (poor, fair, good, or excellent), occupation (agriculture and related workers, factory worker, administrator/manager, professional/technical, sales and service workers, house wife/husband, self-employed, unemployed, retired, or other/not stated), family history of CVD, and jointly stratified by sex, study area, and baseline age in 5-year intervals.

**Table 3:** Hazard ratios of myocardial infarction by PAI.

PAI	Person-Years	Deaths	HR	(95% CI) ^a^	HR	(95% CI) ^b^
Inactive	2,653,654	1226	1.00	(Ref.)	1.00	(Ref.)
≤50	81,979	43	1.05	(0.77–1.42)	1.02	(0.75–1.40)
51–99	67,421	38	0.96	(0.69–1.33)	0.98	(0.71–1.37)
≥100	805,145	501	0.93	(0.83–1.05)	0.94	(0.83–1.05)
<100	2,803,054	1307	1.00	(Ref.)	1.00	(Ref.)
≥100	805,145	501	0.94	(0.84–1.05)	0.93	(0.83–1.05)

CI, confidence interval; HR, hazard ratio; myocardial infarction (ICD10: I21-I23); PAI, Personal Activity Intelligence. ^a^ Adjusted for age as time scale, year of study, and jointly stratified by sex, study area, and baseline age in 5-year intervals. ^b^ Adjusted for age as time scale, year of study, BMI (<18.5, 18.5–24.9, 25–29.9, or ≥30 kg/m^2^), smoking (never, previous, or current), alcohol consumption (average grams of alcohol in a typical week), education (no formal education, primary school, middle or high school, technical school/college, or university), household income, hypertension (yes, no), diabetes status (yes, no), marital status (single, married, widow/er, or separated/divorce), self-rated health (poor, fair, good, or excellent), occupation (agriculture and related workers, factory worker, administrator/manager, professional/technical, sales and service workers, house wife/husband, self-employed, unemployed, retired, or other/not stated), family history of CVD, and jointly stratified by sex, study area, and baseline age in 5-year intervals.

## Data Availability

The CKB study group is committed to making the cohort data available to the scientific community in China, the UK, and worldwide to advance knowledge about the causes, prevention, and treatment of disease. More details about data access, policies, and procedures could be found at the CKB website: https://www.ckbiobank.org/data-access/data-access-procedures (accessed on 1 November 2022).

## Supplementary Materials

The following supporting information can be downloaded at: https://www.mdpi.com/article/10.3390/jcm11216552/s1, Table S1: Baseline characteristics of study participants according to ischemic heart disease status; Table S2: Baseline characteristics of study participants according to myocardial infarction status; Figure S1: Flow of participants in the study.
