# Supplementary material for: Personal Activity Intelligence and Ischemic Heart Disease in a Healthy Population: China Kadoorie Biobank Study
Source: J Clin Med. 2022 Nov 4;11(21):6552. doi: 10.3390/jcm11216552 (PMC9655296; doi:10.3390/jcm11216552)

Supplementary Materials

Hammer et al. Personal Activity Intelligence and Ischemic Heart Disease in a Healthy Population: China Kadoorie Biobank Study

Table S1. Baseline characteristics of study participants according to ischemic heart disease status.

Table S2. Baseline characteristics of study participants according to myocardial infarction status.

Figure S1. Flow of participants in the study.

Table S1. Baseline characteristics of study participants according to ischemic heart disease status

	Ischemic Heart Disease (IHD)							
	**No**				**Yes**			
	Inactive (n=124,676)	≤50 (n=4210)	51-99 (n=3065)	≥100 (n=45,578)	Inactive (n=199,490)	≤50 (n=6183)	51-99 (n=5355)	≥100 (n=55,235)
Age, mean (SD), y	50.4 (10.2)	51.2 (10.2)	51.6 (10.5)	54.8 (10.9)	49.7 (9.9)	50.3 (10.1)	51.2 (10.3)	53.9 (10.2)
Urban residence, No. (%)	45,496 (36.5)	2803 (66.6)	2057 (67.1)	26,516 (58.2)	71,212 (35.7)	4419 (71.5)	2901 (54.2)	37,711 (68.3)
Body mass index, No. (%)								
<18.5	4952 (4.0)	106 (2.5)	63 (2.1)	1668 (3.7)	8589 (4.3)	190 (3.1)	156 (2.9)	1663 (3.0)
18.5-24.9	82,323 (66.0)	2396 (56.9)	1802 (58.8)	28,289 (62.1)	125,609 (63.0)	3763 (60.8)	3354 (62.6)	32,741 (59.3)
25.0-29.9	33,868 (27.2)	1540 (36.6)	1086 (35.4)	14,187 (31.1)	56,522 (28.3)	1905 (30.8)	1594 (29.8)	17,773 (32.2)
≥30.0	3533 (2.8)	168 (4.0)	114 (3.7)	1434 (3.1)	8770 (4.4)	325 (5.3)	251 (4.7)	3058 (5.5)
Systolic BP, mean (SD), mmHg	131.9 (19.6)	131.6 (19.2)	131.5 (19.6)	132.9 (19.9)	129.2 (21.7)	125.8 (21.5)	128.7 (22.5)	129.5 (21.8)
Diastolic BP, mean (SD), mmHg	79.4 (11.4)	79.5 (11.3)	79.0 (11.2)	78.6 (11.2)	76.9 (10.6)	75.3 (10.8)	75.9 (11.2)	76.1 (10.7)
Education level, No. (%)								
No formal education	11,454 (9.2)	99 (2.4)	93 (3.0)	2895 (6.3)	54,561 (27.3)	632 (10.2)	995 (18.6)	9400 (17.0)
Primary school	42,009 (33.7)	800 (19.0)	610 (19.9)	13,428 (29.5)	65,377 (32.8)	1251 (20.2)	1366 (25.5)	15,151 (27.4)
Middle or high school	64,563 (51.8)	2582 (61.3)	1696 (55.3)	23,503 (51.6)	72,960 (36.6)	3476 (56.2)	2523 (47.1)	26,872 (48.7)
College or university	6651 (5.3)	729 (17.3))	666 (21.7)	5752 (12.6)	6592 (3.3)	824 (13.3)	471 (8.8)	3812 (6.9)
Smoking status, No. (%)								
Never	29,821 (23.9)	1356 (32.2)	977 (31.9)	13,898 (30.5)	194,204 (97.4)	6010 (97.2)	5193 (97.0)	53,725 (97.3)
Former	16,545 (13.3)	686 (16.3)	531 (17.3)	8191 (18.0)	1362 (0.7)	59 (1.0)	62 (1.1)	550 (1.0)
Current	78,310 (62.8)	2168 (51.5)	1557 (50.8)	23,489 (51.5)	3924 (1.9)	114 (1.8)	100 (1.9)	960 (1.7)
Regular alcohol intake, No. (%)								
Yes	44,098 (35.4)	1431 (34.0)	1025 (33.4)	14,302 (31.4)	3854 (1.9)	161 (2.6)	117 (2.2)	1263 (2.3)
No	80,578 (64.6)	2779 (66.0)	2040 (66.6)	31,276 (68.6)	195,636 (98.1)	6022 (97.4)	5238 (97.8)	53,972 (97.7)
Household income, yuan/yr, No. (%)[b]								
<10,000	32,757 (26.3)	782 (18.6)	529 (17.3)	10,178 (22.3)	61,082 (30.6)	1263 (20.4)	1742 (32.5)	13,346 (24.1)
10,000-19,999	35,516 (28.5)	1171 (27.8)	849 (27.7)	12,638 (27.7)	59,230 (29.7)	1968 (31.8)	1505 (28.1)	16,219 (29.4)
20,000-34,999	31,067 (24.9)	1173 (27.9)	831 (27.1)	13,038 (28.6)	47,017 (23.6)	1648 (26.7)	1176 (22.0)	15,746 (28.5)
≥35,000	25,336 (20.3)	1084 (25.7)	856 (27.9)	9724 (21.3)	32,161 (16.1)	1304 (21.1)	932 (17.4)	9924 (18.0)
Self-rated health status, No. (%)								
Excellent	26,538 (21.3)	889 (21.1)	780 (25.4)	9910 (21.7)	33,486 (16.8)	1123 (18.2)	795 (14.9)	9495 (17.2)
Good/Fair	89,367 (71.7)	3022 (71.8)	2090 (68.2)	32,941 (72.3)	145,998 (73.2)	4504 (72.8)	3981 (74.3)	41,473 (75.1)
Poor	8771 (7.0)	299 (7.1)	195 (6.4)	2727 (6.0)	20,006 (10.0)	556 (9.0)	579 (10.8)	4267 (7.7)
Family history of CVD, No. (%)								
Yes	24,711 (19.8)	1012 (24.0)	787 (25.7)	9552 (21.0)	38,417 (19.3)	1506 (24.4)	1231 (23.0)	12,179 (22.1)
No	99,965 (80.2)	3198 (76.0)	2278 (74.3)	36,026 (79.0)	161,073 (80.7)	4677 (75.6)	4124 (77.0)	43,056 (77.9)

Table S2. Baseline characteristics of study participants according to myocardial infarction status

	Myocardial Infarction (MI)							
	**No**				**Yes**			
	Inactive (n=124,676)	≤50 (n=4210)	51-99 (n=3065)	≥100 (n=45,578)	Inactive (n=199,490)	≤50 (n=6183)	51-99 (n=5355)	≥100 (n=55,235)
Age, mean (SD), y	50.4 (10.2)	51.2 (10.2)	51.6 (10.5)	54.8 (10.9)	49.7 (9.9)	50.3 (10.1)	51.2 (10.3)	53.9 (10.2)
Urban residence, No. (%)	45,496 (36.5)	2803 (66.6)	2057 (67.1)	26,516 (58.2)	71,212 (35.7)	4419 (71.5)	2901 (54.2)	37,711 (68.3)
Body mass index, No. (%)								
<18.5	4952 (4.0)	106 (2.5)	63 (2.1)	1668 (3.7)	8589 (4.3)	190 (3.1)	156 (2.9)	1663 (3.0)
18.5-24.9	82,323 (66.0)	2396 (56.9)	1802 (58.8)	28,289 (62.1)	125,609 (63.0)	3763 (60.8)	3354 (62.6)	32,741 (59.3)
25.0-29.9	33,868 (27.2)	1540 (36.6)	1086 (35.4)	14,187 (31.1)	56,522 (28.3)	1905 (30.8)	1594 (29.8)	17,773 (32.2)
≥30.0	3533 (2.8)	168 (4.0)	114 (3.7)	1434 (3.1)	8770 (4.4)	325 (5.3)	251 (4.7)	3058 (5.5)
Systolic BP, mean (SD), mmHg	131.9 (19.6)	131.6 (19.2)	131.5 (19.6)	132.9 (19.9)	129.2 (21.7)	125.8 (21.5)	128.7 (22.5)	129.5 (21.8)
Diastolic BP, mean (SD), mmHg	79.4 (11.4)	79.5 (11.3)	79.0 (11.2)	78.6 (11.2)	76.9 (10.6)	75.3 (10.8)	75.9 (11.2)	76.1 (10.7)
Education level, No. (%)								
No formal education	11,454 (9.2)	99 (2.4)	93 (3.0)	2895 (6.3)	54,561 (27.3)	632 (10.2)	995 (18.6)	9400 (17.0)
Primary school	42,009 (33.7)	800 (19.0)	610 (19.9)	13,428 (29.5)	65,377 (32.8)	1251 (20.2)	1366 (25.5)	15,151 (27.4)
Middle or high school	64,563 (51.8)	2582 (61.3)	1696 (55.3)	23,503 (51.6)	72,960 (36.6)	3476 (56.2)	2523 (47.1)	26,872 (48.7)
College or university	6651 (5.3)	729 (17.3))	666 (21.7)	5752 (12.6)	6592 (3.3)	824 (13.3)	471 (8.8)	3812 (6.9)
Smoking status, No. (%)								
Never	29,821 (23.9)	1356 (32.2)	977 (31.9)	13,898 (30.5)	194,204 (97.4)	6010 (97.2)	5193 (97.0)	53,725 (97.3)
Former	16,545 (13.3)	686 (16.3)	531 (17.3)	8191 (18.0)	1362 (0.7)	59 (1.0)	62 (1.1)	550 (1.0)
Current	78,310 (62.8)	2168 (51.5)	1557 (50.8)	23,489 (51.5)	3924 (1.9)	114 (1.8)	100 (1.9)	960 (1.7)
Regular alcohol intake, No. (%)								
Yes	44,098 (35.4)	1431 (34.0)	1025 (33.4)	14,302 (31.4)	3854 (1.9)	161 (2.6)	117 (2.2)	1263 (2.3)
No	80,578 (64.6)	2779 (66.0)	2040 (66.6)	31,276 (68.6)	195,636 (98.1)	6022 (97.4)	5238 (97.8)	53,972 (97.7)
Household income, yuan/yr, No. (%)[b]								
<10,000	32,757 (26.3)	782 (18.6)	529 (17.3)	10,178 (22.3)	61,082 (30.6)	1263 (20.4)	1742 (32.5)	13,346 (24.1)
10,000-19,999	35,516 (28.5)	1171 (27.8)	849 (27.7)	12,638 (27.7)	59,230 (29.7)	1968 (31.8)	1505 (28.1)	16,219 (29.4)
20,000-34,999	31,067 (24.9)	1173 (27.9)	831 (27.1)	13,038 (28.6)	47,017 (23.6)	1648 (26.7)	1176 (22.0)	15,746 (28.5)
≥35,000	25,336 (20.3)	1084 (25.7)	856 (27.9)	9724 (21.3)	32,161 (16.1)	1304 (21.1)	932 (17.4)	9924 (18.0)
Self-rated health status, No. (%)								
Excellent	26,538 (21.3)	889 (21.1)	780 (25.4)	9910 (21.7)	33,486 (16.8)	1123 (18.2)	795 (14.9)	9495 (17.2)
Good/Fair	89,367 (71.7)	3022 (71.8)	2090 (68.2)	32,941 (72.3)	145,998 (73.2)	4504 (72.8)	3981 (74.3)	41,473 (75.1)
Poor	8771 (7.0)	299 (7.1)	195 (6.4)	2727 (6.0)	20,006 (10.0)	556 (9.0)	579 (10.8)	4267 (7.7)
Family history of CVD, No. (%)								
Yes	24,711 (19.8)	1012 (24.0)	787 (25.7)	9552 (21.0)	38,417 (19.3)	1506 (24.4)	1231 (23.0)	12,179 (22.1)
No	99,965 (80.2)	3198 (76.0)	2278 (74.3)	36,026 (79.0)	161,073 (80.7)	4677 (75.6)	4124 (77.0)	43,056 (77.9)

Figure S1. Flow of participants in the study.

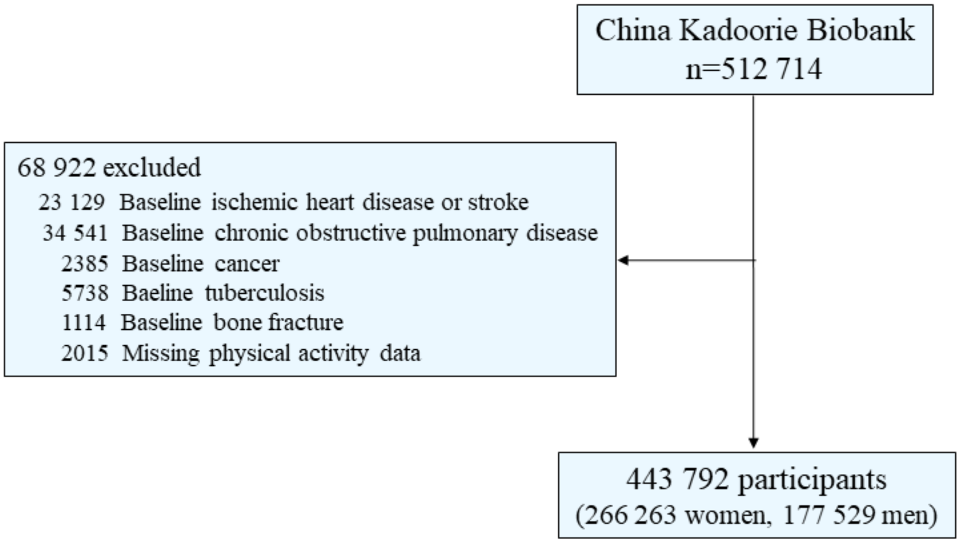

Supplement: Supplementary file 1 [file jcm-11-06552-s001.zip › jcm-1942833-supplementary.pdf]
